# Supplementary material for: Progress toward closing gaps in the hepatitis C virus cascade of care for people who inject drugs in San Francisco
Source: PLoS One. 2021 Apr 2;16(4):e0249585. doi: 10.1371/journal.pone.0249585 (PMC8018615; doi:10.1371/journal.pone.0249585)
Supplement: S1 Fig — Denominators for percent who received treatment and attained sustained virologic response (SVR) are those diagnosed with HCV infection. In 2015, tested antibody positive and attained SVR were not measured. The estimates in this graph are not RDS-adjusted (i.e., crude). (DOCX) [file pone.0249585.s001.docx]

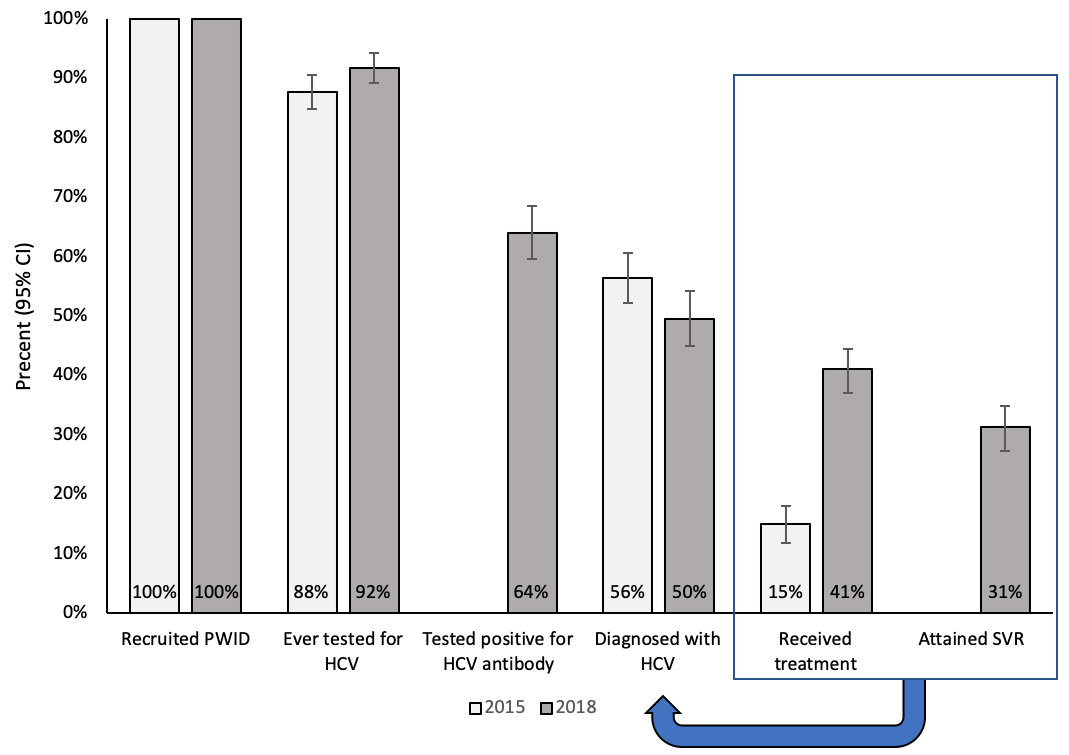


**Supplement Figure 1**. Crude estimates for the Hepatitis C virus (HCV) infection treatment cascade in two rounds of surveys of people who inject drugs (PWID) in San Francisco, 2015 and 2018. Denominators for percent who received treatment and attained sustained virologic response (SVR) are those diagnosed with HCV infection. In 2015, tested antibody positive and attained SVR were not measured. The estimates in this graph are **not** RDS-adjusted (i.e., crude).
